# Characteristics of optic disc hemorrhage and optic nerve changes following acute primary angle closure

**DOI:** 10.3389/fneur.2024.1333091

**Published:** 2024-05-24

**Authors:** Hengli Zhang, Yawen Li, Yizhen Tang, Xiaowei Yan, Yulei Geng, Weijia Li, Kuitang Shi, Guangxian Tang, Hongtao Guo

**Affiliations:** ^1^Department of Ophthalmology, Shijiazhuang People’s Hospital, Shijiazhuang, China; ^2^Beijing Ophthalmology and Visual Sciences Key Laboratory, Department of Ophthalmology, Beijing Tongren Hospital, Capital Medical University, Beijing, China; ^3^Department of Orthopedics, Shijiazhuang People’s Hospital, Shijiazhuang, China

**Keywords:** acute primary angle closure, intraocular pressure, optic disc hemorrhage, retinal nerve fiber layer, visual field

## Abstract

**Introduction:**

Acute primary angle closure (APAC) is an emergency ophthalmic presentation and a major cause of irreversible blindness in China. However, only a few studies have focused on the characteristics of optic disc hemorrhage (ODH) during an APAC attack, including its shape, depth, location, scope, and duration after intraocular pressure (IOP) control, along with changes in the optic nerve. This study aimed to analyze the characteristics of ODH and optic nerve changes in patients during their first APAC episode.

**Methods:**

This retrospective study involved 32 eyes from 32 patients with APAC who received sequential treatment and analyzed the following parameters: the highest IOP and its duration, ODH, retinal nerve fiber layer thickness (RNFLT), and mean deviation (MD). We compared parameters obtained from the affected eye (ODH group) and contralateral unaffected eye (control group), as well as intragroup comparisons.

**Results:**

The mean IOP in the ODH group was 64.28 ± 10.36 mmHg, with a duration of 4.44 ± 2.35 days. Flame and splinter shapes accounted for 84.38% of the ODH. The mean ODH duration was 4.81 ± 3.25 weeks. ODH during APAC was isolated to one sector in 59.38% of cases, mostly occurring in the temporal superior and temporal inferior (each accounting for 21.88% of the cases). There was a positive correlation between the extent of hemorrhage and the highest IOP duration (*p* < 0.001). RNFLT was significantly thickened within 72 h post-IOP control but was thinned by 2 weeks. By 6 months, the thinning stabilized, and there was no difference noted between the ODH and control groups at 12 months. MD partly improved at 6 months post-IOP control, and ODH scope significantly affected the MD (*p* < 0.001). The duration of high IOP was positively correlated to the ODH scope and MD damage.

**Discussion:**

Timely and effective IOP management is essential for recovering visual function following an APAC attack.

## 1 Introduction

Primary angle closure glaucoma (PACG), a serious and irreversible blinding eye disease, is the main type of glaucoma across Asia, with approximately 48% of affected patients based in China (1, 2). Acute primary angle closure (APAC) is an emergency ophthalmic presentation and a major cause of irreversible blindness in China (3, 4). It is characterized by a sudden increase in intraocular pressure (IOP), reduced visual acuity (VA), and ocular pain, eventually leading to severe visual impairment and glaucomatous optic neuropathy development (5). However, high IOP has diverse effects on the optic nerve during APAC attacks (6–8). Studies have reported a high prevalence of APAC in Asian populations, often presenting with more severe and longer duration (1, 9).

Prolonged elevation of IOP mechanically compresses the optic nerve, disrupting the lamina cribrosa continuity, distorting lamina cribrosa pores, impeding axoplasmic flow, and causing optic disc hemorrhage (ODH) (10–12). However, only a few studies have investigated the characteristics of ODH during an APAC attack. Accordingly, the shape, depth, location, scope, and duration of ODH after IOP control, as well as optic nerve changes, remain largely unclear. In this study, we aimed to analyze ODH characteristics and optic nerve changes following an initial APAC attack to reveal their impact on disease progression and prognosis.

## 2 Materials and methods

### 2.1 Patients

In this retrospective study, 32 patients (32 eyes) with ODH during an APAC attack who were admitted to Shijiazhuang People’s Hospital between October 2018 and January 2020 were recruited. All cases presented with a single-eye attack. In each patient, the eye experiencing the acute attack with the highest initial IOP and ODH was categorized into the ODH group, whereas the contralateral unaffected eye, maintaining a controlled IOP and without ODH, served as the control group. All patients underwent routine ophthalmic examinations when the cornea was transparent.

The highest IOP and its duration, ODH characteristics, best-corrected visual acuity (BCVA), RNFLT, and VF were recorded within 72 h and at 2 weeks, 1 month, 3 months, 6 months, and 12 months after IOP control. This study adhered to the principles of the Declaration of Helsinki and received approval from the Ethics Committee of Shijiazhuang People’s Hospital. All patients provided written informed consent.

### 2.2 Eligibility

This study included patients who met the following criteria: (1) aged >18 years; (2) diagnosed using the International Society of Geographic and Epidemiologic Ophthalmology diagnostic criteria (3, 13); (3) experiencing their first APAC attack with no history of intraocular surgery; (4) presenting with eye or periorbital pain, accompanied by ipsilateral headache, nausea, decreased vision, conjunctival congestion, corneal epithelial edema, shallow anterior chamber, mydriasis, and light reflex disappearance; and (5) exhibiting angle closure under dynamic gonioscopy.

The exclusion criteria were as follows: (1) secondary angle closure, such as uveitis and neovascular glaucoma; (2) APAC with a confirmed diagnosis of PACG; (3) multiple APAC attacks; (4) history of intraocular surgery; (5) unreliable OCT and VF examination results; (6) exceeding ±5.0 D and ± 3.0 D refractive astigmatism; and (7) presence of retinopathy or other conditions that damage the optic nerve, such as diabetes and hypertension.

### 2.3 Routine ophthalmic examination

A Snellen chart was used for BCVA assessment, with results expressed using the logarithmic minimum angle of resolution (logMAR). IOP was measured using a calibrated Goldmann applanation tonometer. Gonioscopy, with a single mirror Gonio diagnostic lens, was used to measure the anterior chamber angle, whereas the anterior segment was examined using a slit-lamp microscope. Direct ophthalmoscopic examination and stereophotography were conducted to detect ODH. All examinations were performed by experienced ophthalmologists and technicians.

### 2.4 ODH classification and examination

**Figure 1:**
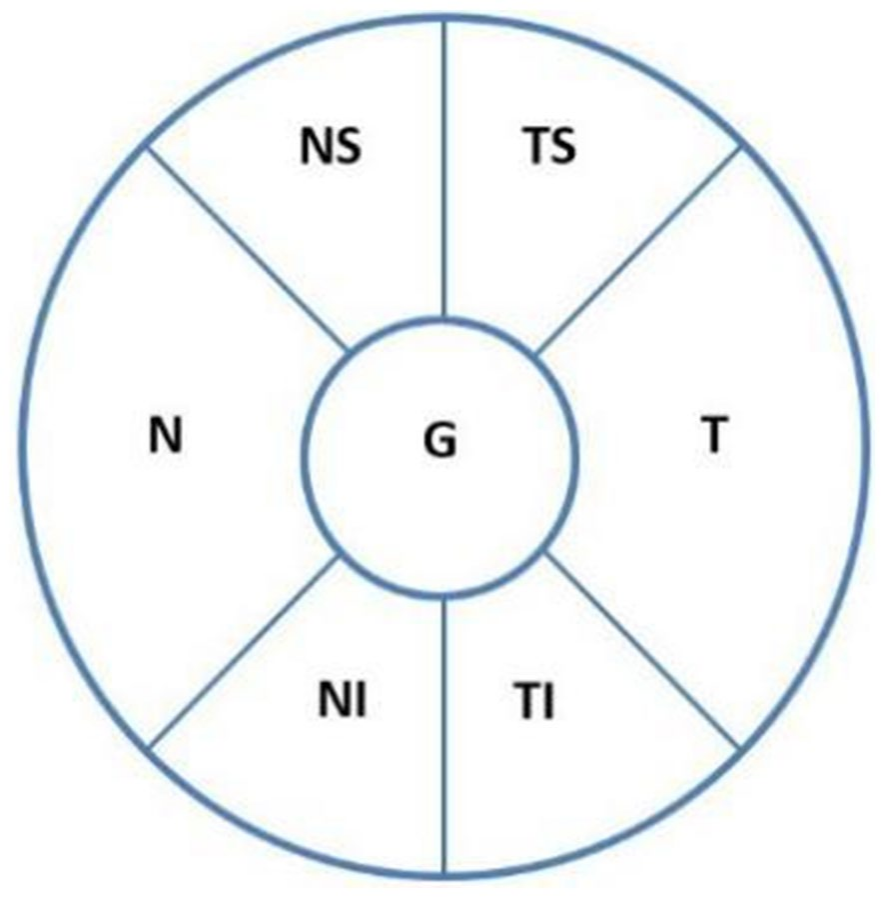
ODH locations according to the optic disc, as revealed on SD-OCT automatic scanning. The sectors are as follows: temporal superior (TS), temporal (T), temporal inferior (TI), nasal superior (NS), nasal (N), and nasal inferior (NI). ODH, optic disc hemorrhage.

ODH was defined as a hemorrhage within one disc diameter of the optic nerve (14) and presented in a splinter, flame, or linear shape radially perpendicular to the optic disc edge or as parapapillary hemorrhage (<1 papilla diameter) (15). Using spectral-domain optical coherence tomography (SD-OCT; Spectralis OCT, Heidelberg, Germany) automatic scanning (Figure 1), the ODH scope, which is the extent of ODH distribution within the optic disc, was divided into different sectors as follows: temporal superior (TS), temporal (T), temporal inferior (TI), nasal superior (NS), nasal (N), and nasal inferior (NI). Within 3 months after IOP control, ODH changes were closely monitored using direct ophthalmoscopy and stereo photography (Kowa Nonmyd WX-3D) every 2 weeks, with stereoscopic optic disc imaging.

### 2.5 VF examination

The Humphrey-750i Field Analyzer (Carl Zeiss Meditec, Oberkochen, Germany) was used to assess the VF. Before VF testing, the patients underwent slit-lamp and fundus examinations to exclude factors that may influence VF examination, such as corneal abnormalities or retinal and optic neuropathies. The reliability criteria comprised a fixation loss rate of <20% and false-negative and false-positive rates of <15%. The mean deviation (MD) was recorded based on the results of three tests conducted to establish a reliable baseline, and unreliable VFs were examined repeatedly. VF and OCT were performed by experienced doctors within the same month.

### 2.6 OCT examination

The SD-OCT (Spectralis OCT) optic disc examination was performed with the optic disc at the center and a diameter of 3.4 mm for circular scanning. The measurement parameters were as follows: TS, T, TI, NS, N, NI, the average RNFLT (G), and the depth of the ODH.

### 2.7 Statistical analysis

All data were analyzed using SPSS 20.0 (IBM, Armonk, NY, United States). The measurement values are expressed as mean ± standard deviation, where n represents the number of eyes. The parameters of the ODH and control groups at the same time point were compared using paired *t*-tests. Repeated measures analysis of variance was used to detect intragroup variations of the RNFLT and MD at different time points after IOP control, and the Student–Newman–Keuls test was used to compare intragroup differences between two time points. Linear regression analysis was used to assess the association between the highest IOP, its duration, and ODH scope, as well as between the ODH scope and MD. *p* < 0.05 was considered statistically significant.

## 3 Results

The patients were aged between 45 and 74 years (65.13 ± 6.91) and included 8 men (8 eyes) and 24 women (24 eyes).

### 3.1 APAC treatment measures

In the ODH group, 28 eyes underwent trabeculectomy, 2 underwent vitreous aspiration and anterior chamber reformation, and 2 underwent phacoemulsification combined with goniosynechialysis. In the control group, surgical and laser peripheral iridectomy procedures were performed in 18 and 14 eyes, respectively.

### 3.2 IOP

The average duration of increased IOP during the APAC attack was 4.44 ± 2.35 (range 2–11) days. In the ODH group, the mean IOP during the APAC attack was 64.28 ± 10.36 mmHg (range 56–87 mmHg), which was significantly higher than that of the control group (16.66 ± 2.31 mmHg; *t* = 25.64, *p* < 0.001). At 12 months post-IOP control, the mean IOP in the ODH and control groups was 16.38 ± 2.77 and 15.56 ± 1.76 mmHg, respectively, with no statistically significant difference observed (*t* = 1.34, *p* = 0. 19). IOP was maintained at the target IOP level in all patients during the follow-up period. At 6 months post-IOP control, two patients with elevated IOP in the ODH group received an IOP-lowering medication, whereas another patient was treated with two IOP-lowering medications to achieve an IOP < 21 mmHg. The changes in IOP during the follow-up period are shown in Table 1.

**Table 1:** Changes in IOP (mmHg) before and after IOP control in the ODH and control groups.

	ODH group	Control group	*t*	*p*
Initial visit	64.28 ± 10.36	16.66 ± 2.31	25.64	<0.001
2 weeks after IOP control	14.81 ± 2.73	15.81 ± 2. 12	1.86	0.07
1 month after IOP control	14.94 ± 3.00	15.52 ± 2.26	0.77	0.45
3 months after IOP control	15.61 ± 3.22	15.36 ± 1.99	0.40	0.69
6 months after IOP control	16.03 ± 3.40	15.45 ± 1.71	0.88	0.39
12 months after IOP control	16.38 ± 2.77	15.56 ± 1.76	1.34	0.19

ODH, optic disc hemorrhage; IOP, intraocular pressure.

### 3.3 BCVA

In the ODH group, the mean 72 h post-IOP control BCVA was 1.48 ± 0.42, which differed significantly from that of the control group (*t* = 16.53, *p* < 0.001). The BCVA improved after IOP control, demonstrating a significant intragroup difference (*F* = 159.4, *p* < 0.001). There was a significant improvement of BCVA at 2 weeks post-IOP control compared to that within 72 h post-IOP control (*q* = 22.42, *p* < 0.001); this improvement was also observed at 1 month compared to that at 2 weeks (*q* = 5.69, *p* < 0.001). However, no significant alteration was observed at 6 months post-IOP control compared to that at 1 month post-IOP control (*q* = 1.65, *p* > 0.05) (Table 2).

**Table 2:** Changes in BCVA following IOP control in the ODH and control groups according to LogMAR.

	ODH group	Control group	*t*	*p*
Within 72 h of IOP control	1.48 ± 0.42	0.21 ± 0.16	16.53	<0.001
2 weeks post-IOP control	0.66 ± 0.22a	0.21 ± 0.16	9.27	<0.001
1 month post-IOP control	0.45 ± 0. 18b	0.21 ± 0.16	5.79	<0.001
3 months post-IOP control	0.40 ± 0.18	0.21 ± 0.15	4.54	<0.001
6 months post-IOP control	0.39 ± 0.15	0.22 ± 0.15	4.54	<0.001

ODH, optic disc hemorrhage; BCVA, best-corrected visual acuity; LogMAR, logarithmic minimum angle of resolution; APAC, acute primary angle-closure; ANOVA, analysis of variance.

Compared to 72 h post-IOP control, ^a^*p* < 0.001, repeated-measures ANOVA.

Compared to 2 weeks post-IOP control. ^b^*p* < 0.001, repeated-measures ANOVA.

### 3.4 ODH

**Figure 2:**
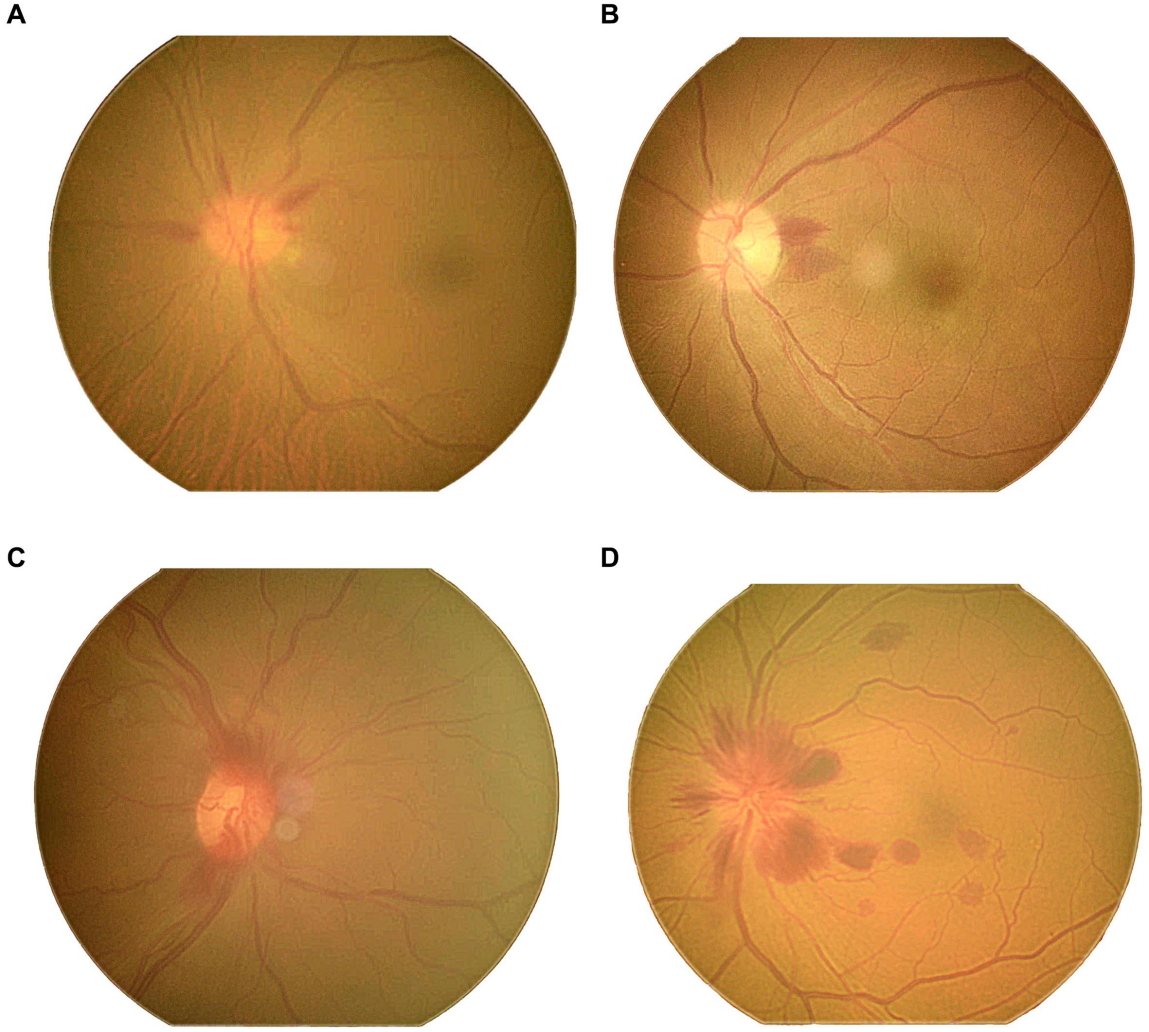
Photos of the different types of ODH: **(A)** linear ODH shapes; **(B)** splinter ODH shapes; **(C,D)** flame ODH shapes.

The mean ODH duration was 4.81 ± 3.25 (range: 2–13) weeks. Flame and splinter ODH shapes constituted 84.38% (27 eyes) of all cases, whereas linear shapes accounted for 15.63% (5 eyes). The hemorrhages were located in the superficial layer of the retina; 62.5% (20 eyes) of the hemorrhages were radially perpendicular to the optic disc edge, whereas 37.5% (12 eyes) were parapapillary hemorrhages. Moreover, 12.5% (4 eyes) of the cases were accompanied by a flame-shaped hemorrhage along the blood vessels to the peripheral retina (Figure 2).

**Figure 3:**
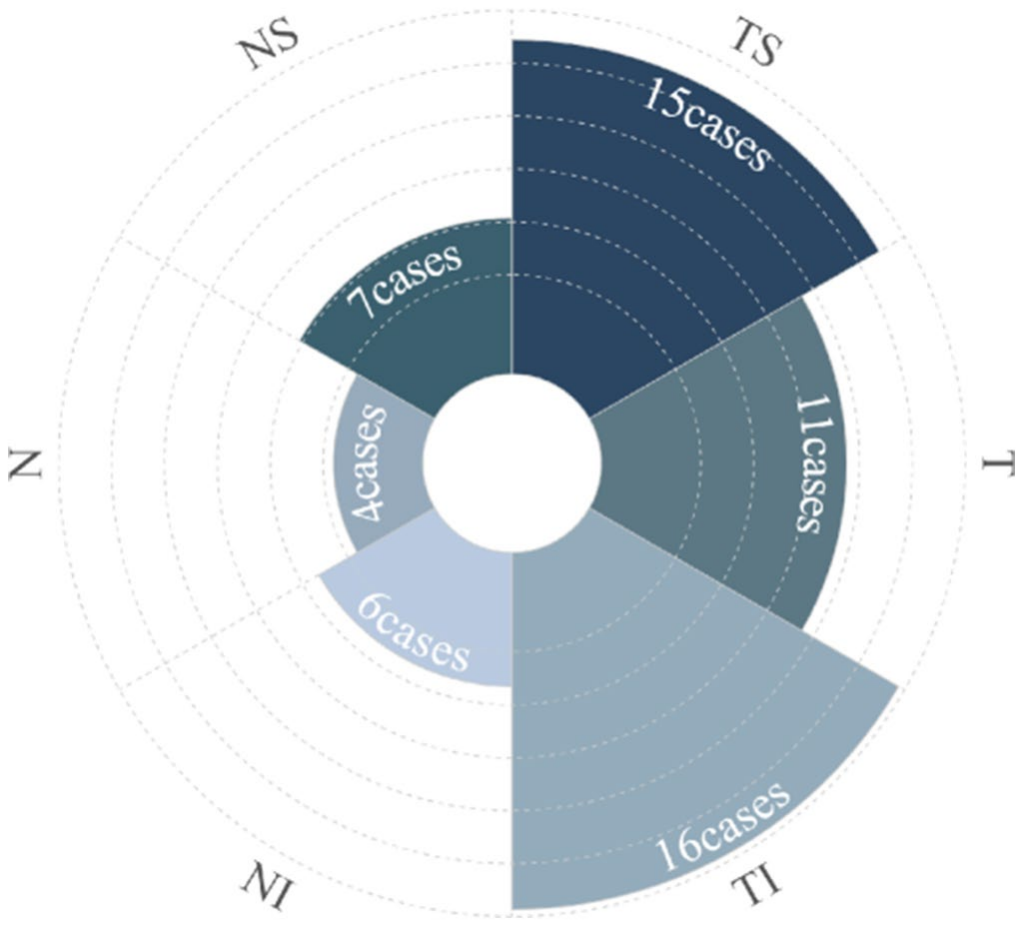
The polar diagram shows the number of cases with disc hemorrhages in relationship to ON sectors.

ODH was isolated to one sector in 59.38% (19 eyes); TS, TI, and T accounted for 21.88% (7 eyes), 21.88% (7 eyes), and 15.63% (5 eyes), respectively. Hemorrhages spanned two, three, and four sectors, and the circle accounted for 18.75% (6 eyes), 12.5% (4 eyes), 3.13% (1 eye), and 6.25% (2 eyes), respectively. Among the 32 cases in the ODH group, the overall prevalence of ODH in the TI, TS, T, NS, NI, and N sectors was 50% (16 cases), 46.88% (15 cases), 34.37% (11 cases), 21.88% (7 cases), 18.75% (6 cases), and 12.5% (4 cases), respectively (Figure 3).

### 3.5 RNFLT

#### 3.5.1 Comparison of RNFLT at different time points between both groups

In the ODH group, the RNFLT in each sector at 72 h post-IOP control was significantly thicker than that of the control group (all *p* < 0.001). With the exception of the temporal side (*p* = 0.92), the RNFLT in the other sectors remained significantly thickened at 2 weeks (all *p* < 0.001). However, it was significantly thinner in each sector from 1 to 12 months of follow-up post-IOP control (all *p* < 0.05) (Table 3).

**Table 3:** Changes in RNFLT (μm) following IOP control in the ODH and control groups.

	ODH group	Control group	*t*	*p*
**Within 72 h post-IOP control**				
NS	187.93 ± 53.76	120.50 ± 15.63	7.37	<0.001
N	126.71 ± 32.24	83.70 ± 9.52	5.84	<0.001
NI	202. 14 ± 41.58	129.05 ± 16.21	9.68	<0.001
TS	232.71 ± 33.04	144.65 ± 15.48	14. 14	<0.001
T	112.86 ± 37.16	82.65 ± 12.95	4.15	<0.001
TI	267.29 ± 43.33	150.05 ± 18.51	13.97	<0.001
G	157.29 ± 21.08	105.00 ± 6.46	12.96	<0.001
**2 weeks post-IOP control**				
NS	151.07 ± 42.73	117.95 ± 16.47	3.78	<0.001
N	108. 14 ± 25.05	82.30 ± 10.53	3.82	<0.001
NI	162.43 ± 31.36	128.05 ± 15.50	6.23	<0.001
TS	170.28 ± 24.78	142.80 ± 13.77	6.27	<0.001
T	84.36 ± 17.42	81.75 ± 12.08	0.095	0.92
TI	185.07 ± 28.97	148.95 ± 19.17	6.18	<0.001
G	131.71 ± 14.30	104.44 ± 6.77	10.84	<0.001
**1 month post-IOP control**				
NS	107.5 ± 26.19	118.40 ± 15.31	2.91	<0.01
N	79.29 ± 21.53	82.25 ± 9.90	2.09	0.05
NI	114.71 ± 26.06	128.00 ± 15.44	2. 12	0.04
TS	113.36 ± 24.26	142.65 ± 14.80	5.99	<0.001
T	61.93 ± 15.95	81.85 ± 12.55	5.98	<0.001
TI	122.00 ± 38.01	148.95 ± 19.17	3.48	<0.001
G	93.50 ± 19.97	103.95 ± 6.81	3.06	<0.001
**3 months post-IOP control**				
NS	76.93 ± 26.06	117. 10 ± 16.61	8.27	<0.001
N	63.93 ± 22.63	82.20 ± 8.80	5.82	<0.001
NI	114.71 ± 26.06	127.95 ± 15.39	5.74	<0.001
TS	92.57 ± 21.08	141.30 ± 14.56	11.01	<0.001
T	55.57 ± 12.64	79.70 ± 11.76	9.02	<0.001
TI	94.79 ± 35.85	147.80 ± 18.03	7.67	<0.001
G	74.36 ± 21.15	103.60 ± 7.04	7.63	<0.001
**6 months post-IOP control**				
NS	66.71 ± 23.19	117.25 ± 16.24	11. 1	<0.001
N	58.71 ± 24.68	81.90 ± 10.07	6.49	<0.001
NI	89.86 ± 29.36	127.50 ± 15.69	8.20	<0.001
TS	79.79 ± 24.28	142.30 ± 12.43	12.72	<0.001
T	52.00 ± 13.13	80.65 ± 11.91	9.70	<0.001
TI	80.57 ± 30.45	147.30 ± 18.81	12.51	<0.001
G	64.50 ± 19.19	103.70 ± 6.29	11.2	<0.001
**12 months post-IOP control**				
NS	66.07 ± 23.18	118.45 ± 16.57	10.74	<0.001
N	57.00 ± 22.69	83.55 ± 9.59	7.32	<0.001
NI	78.64 ± 26.89	127. 15 ± 16.46	9.95	<0.001
TS	78.50 ± 21.96	143.20 ± 12.30	13.60	<0.001
T	51.21 ± 11.99	80.45 ± 12.20	9.74	<0.001
TI	80.43 ± 31.58	145.85 ± 17.08	12.57	<0.001
G	65.50 ± 19.93	103.40 ± 6.20	10.33	<0.001

ODH, optic disc hemorrhage; RNFLT, retinal nerve fiber layer thickness; TS, temporal superior; T, temporal; TI, temporal inferior; NS, nasal superior; N, nasal; NI, nasal inferior; G, the average RNFLT; IOP, intraocular pressure.

#### 3.5.2 Comparison of RNFLT at different time points in the control group

**Figure 4:**
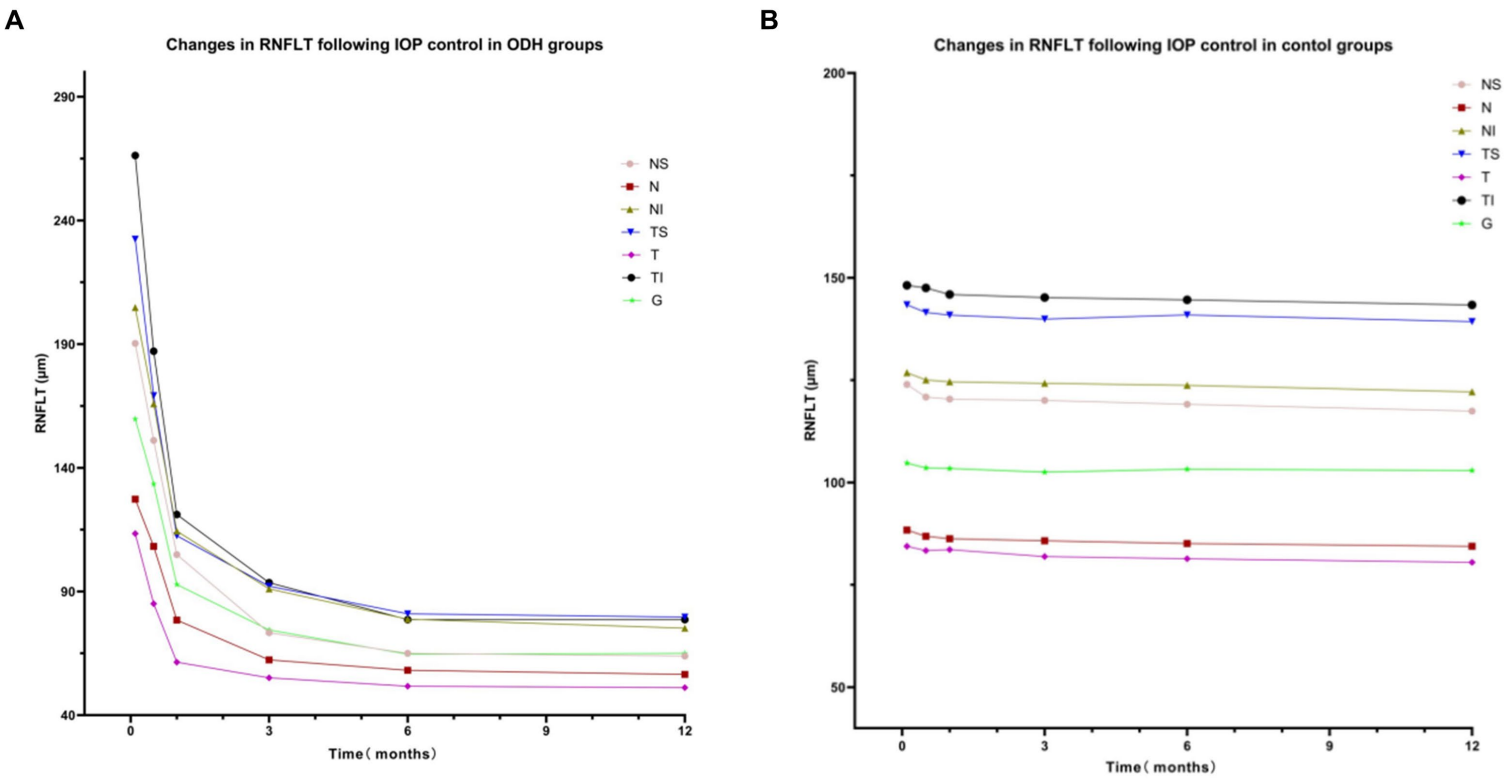
Changes in RNFLT following IOP control in the ODH **(A)** and control **(B)** groups.

In the control group, no significant difference in RNFLT was observed in any sector during the follow-up period (NS: *F* = 0.75, *p* = 0.59; N: *F* = 0.71, *p* = 0.62; NI: *F* = 0.27, *p* = 0.93; TS: *F* = 0.33, *p* = 0.90; T: *F* = 0.58, *p =* 0.72; TI: *F* = 0.30, *p* = 0.91; G: *F* = 0.35, *p* = 0.88; Figure 4).

#### 3.5.3 Comparison of RNFLT at different time points in the ODH group

In the ODH group, RNFLT decreased with time (NS: *F* = 97.36, *p* < 0.001; N: *F* = 81.39, *p* < 0.001; NI: *F* = 142.2, *p* < 0.001; TS: *F* = 289.6 *p* < 0.001; T: *F* = 63.56, *p* < 0.001; TI: *F* = 188.4, *p* < 0.001; G: *F* = 199.2, *p* < 0.001). At 2 weeks, no significant difference in RNFLT was observed in the nasal sector (*p* > 0.05). However, the RNFLT at all other time points was significantly thinner (all *p* < 0.05) than at 72 h post-IOP control. The RNFLT in each sector was thinner after 1 month than at 2 weeks post-IOP control (*p* < 0.001). No significant difference in temporal RNFLT was observed between 1 and 3 months post-IOP control (*p* > 0.05), whereas RNFLT in the other sectors decreased (*p* < 0.05). At the same time, RNFLT in the TS, TI, and G sectors was significantly lower at 6 months post-IOP control than at 3 months post-IOP control (*p* < 0.05); no significant difference was observed in the other sectors (*p* > 0.05). RNFLT in each sector within 1 year after IOP control showed no significant change compared to 6 months after IOP control (*p* > 0.05). The post-IOP control changes in RNFLT in the ODH group are shown in Figure 4.

#### 3.5.4 Changes in RNFLT at different time points and hemorrhage locations in the ODH group

The RNFLT gradually thinned in ODH located in TS and TI (TS: *F* = 159.9, *p* < 0.001; TI: *F* = 202.6, *p* < 0.001), and was significantly thinner at 6 months post-IOP control (both *p* < 0.05). No significant changes were observed between 6 and 12 months post-IOP control (*p* > 0.05). Intragroup analysis revealed that during the follow-up period, RNFLT in the T sector at each time point differed significantly (*F* = 127.3, *p* < 0.001), with a significant reduction in RNFLT at 2 weeks and 1-month post-IOP control compared to that at 72 h post-IOP control (*p* < 0.01). No significant difference was observed at each time point within 3 months post-IOP control (*p* > 0.05) (Table 4).

**Table 4:** Changes in RNFLT (μm) following IOP control in the ODH group.

	TS (*n* = 15)	TI (*n* = 16)	T (*n* = 11)	NS (*n* = 7)	NI (*n* = 6)	N (*n* = 4)
Within 72 h post-IOP control	247.78 ± 31.3	287.4 ± 28.20	147.80 ± 38.36	232.75 ± 61.17	239.30 ± 68.19	154.7 ± 17.62
2 weeks post-IOP control	179.56 ± 22.28	178.90 ± 35.22	97.80 ± 24.58	188.25 ± 64. 11	181.30 ± 38.94	111.70 ± 17.10
1 month post-IOP control	121.44 ± 20.92	107.00 ± 42.07	55.60 ± 21.73	118.25 ± 27.09	128.0 ± 19.29	66.67 ± 15.50
3 months post-IOP control	95.88 ± 24.97	76.63 ± 25.37	49.40 ± 13.54	58.25 ± 20.84	98.67 ± 27.29	46.33 ± 16.29
6 months post-IOP control	79.78 ± 29.24	63.75 ± 18.93	46.80 ± 12.13	45.00 ± 5.72	82.67 ± 21.22	45.33 ± 22.23
12 months post-IOP control	78.78 ± 27.64	62.25 ± 19.94	45.20 ± 12.23	45.50 ± 6.76	82.33 ± 20.11	46.00 ± 20.78

RNFLT, retinal nerve fiber layer thickness; TS, temporal superior; T, temporal; TI, temporal inferior; NS, nasal superior; N, nasal; NI, nasal inferior; G, the average RNFLT; ODH, optic disc hemorrhage; IOP, intraocular pressure.

**Figure 5:**
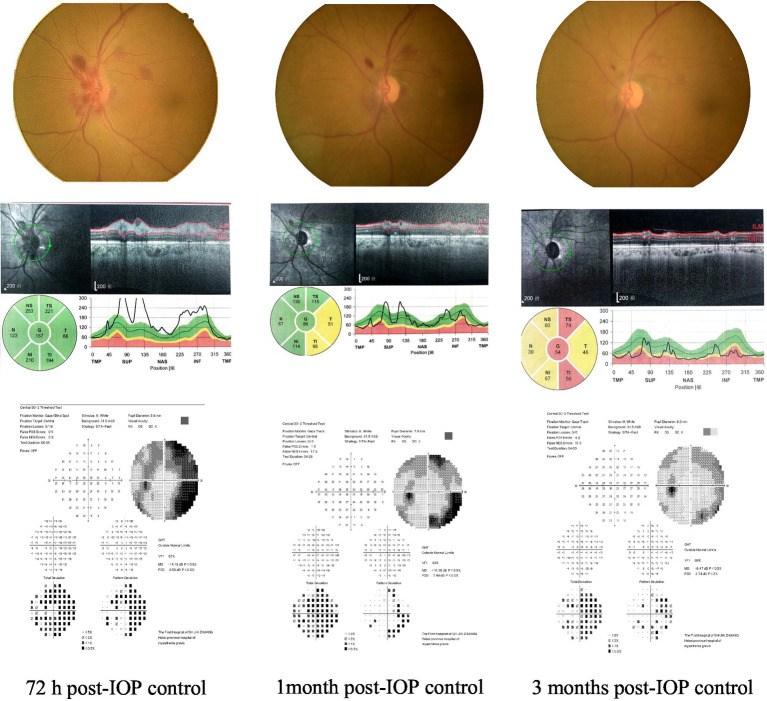
A case: changes in ODH, MD, and RNFLT at 72 h, 1 month, and 3 months after IOP control.

Within the ODH group, RNFLT in the hemorrhages located in TS, TI, and T increased at 72-h post-IOP control; however, the differences were not statistically significant (*p* = 0.18, 0.14, and 0.06, respectively). No distinct differences were observed at 2 weeks, 1 month, 3 months, 6 months, and 12 months following IOP control (all *p* > 0.05). RNFLT data from other sectors (NS: 7 eyes; N: 4 eyes; NI: 6 eyes) were not statistically analyzed owing to the small sample sizes (Table 4). Figure 5 shows a representative case.

### 3.6 MD

The MD at each time point post-IOP control in the ODH group was significantly lower than that in the control group (all *p* < 0.001); however, it increased gradually over time (*F* = 63.65, *p* < 0.001). The MD significantly increased at 1-month post-IOP control compared to 72 h (*p* < 0.001). Within the ODH group, significant differences were observed at all time points from 1 to 6 months post-IOP control (all *p* < 0.05), whereas no significant difference was observed from 6 to 12 months (*p >* 0.05). No significant changes in MD over time were observed in the control group (*F* = 2.33, *p* = 0.07) (Table 5).

**Table 5:** Changes in the MD (dB) in the two groups.

	ODH group	Control group	*t*	*p*
Within 72 h post-IOP control	−14.41 ± 5.75	−1.37 ± 1.21	13.33	<0.001
1 month post-IOP control	−10.53 ± 4.36	−1.36 ± 1.04	13.03	<0.001
3 months post-IOP control	−8.63 ± 3.94	−1.49 ± 1.05	11.74	<0.001
6 months post-IOP control	−6.95 ± 4.37	−1.58 ± 1.04	7.48	<0.001
12 months post-IOP control	−6.93 ± 3.08	−1.59 ± 1.16	9.89	<0.001

ODH, optic disc hemorrhage; MD, mean deviation; IOP, intraocular pressure.

### 3.7 Regression and correlation analyses

#### 3.7.1 Relationship between the highest IOP, its duration, and ODH scope

Multiple linear regression analysis revealed a statistically significant association (*F* = 72.54, *p* < 0.001), with the duration of the highest IOP exhibiting a significant positive effect on ODH scope (*β* = 0.53, *t* = 9.56, *p* < 0.001); the longer the duration, the greater the scope. However, no significant correlation was observed between the highest IOP and ODH scope (*β* = 0.00, *t* = −0.35, *p* = 0.97).

#### 3.7.2 Correlation between ODH scope and the MD after 12 months of IOP control

Linear regression analysis revealed that the regression model was statistically significant (*F* = 79.23, *p* < 0.001) and that the ODH scope significantly impacted MD value (*β* = 1.97, *T* = −8.90, *p* < 0.001); the larger the scope, the more severe the damage.

## 4 Discussion

ODH occurs in 57.5% of primary open-angle glaucoma (POAG) cases, 37.5% of normal-tension glaucoma (NTG) (14) cases, and approximately 5% of PACG cases, with lower rates in Asia (16). For POAG and NTG, ODH often indicates disease progression, with corresponding RNFLT thinning and atrophy after hemorrhage absorption. Disease progression can be effectively controlled by reducing IOP (12, 15). Lan et al. (16) reported that ODH in PACG was associated with the progression of optic atrophy and VF defects. However, investigations focusing on ODH in PACG, particularly in APAC, are limited.

This study revealed that ODH predominantly occurred as flame- and splinter-shaped hemorrhages during the first acute attack of APAC (84.38%), located in the superficial layer of the retina, primarily in the TI and TS sectors. The single-sector ODH occurrence rates were 21.88% each in the TI and TS sectors and 15.63% in the T sector, whereas the overall ODH occurrence rate was 50, 46.88, and 34.37% in the TI, TS, and T sectors, respectively. These findings align with the results of previous studies on POAG, ocular hypertension, and NTG disc hemorrhage (14, 17, 18). The mean ODH duration was 4.81 ± 3.25 weeks, similar to that reported by Lan et al. (16). These findings indicate that ODH in APAC attacks mostly occurs at the TI and TS sectors, possibly due to prolonged high IOP exerting pressure on the optic disc, lamina cribrosa (LC), and LC pores. This pressure eventually leads to microvascular ischemia, autoregulatory dysfunction, vascular dysregulation, and axoplasmic transport obstruction in the LC area. This can also influence axonal physiological function and nutrition, causing continuous LC interruption, LC pore deformation, and eventually capillarization through the ruptured area (11, 12). Alternatively, RNFLT in the TI and TS sectors of the optic disc may be thicker because of peak RNFLT distribution along the temporal vascular branches of the retina. Hence, under the same conditions (e.g., elevated IOP), a thicker RNFLT is more likely to be involved in ODH (17, 19).

The present study also found that four eyes (12.5%) were simultaneously accompanied by a flame-shaped hemorrhage along the blood vessels to the peripheral retina—namely, a decompression retinopathy. The highest IOP values recorded in these patients were 81, 78, 87, and 52 mmHg, with highest IOP durations of 10, 11, 8, and 6 days and hemorrhage durations of 12, 13, 10, and 4 weeks, respectively. These patients were otherwise healthy with no history of other diseases. Similar to our results, Nah et al. reported that decompression retinopathy occurred after the IOP decreased in patients experiencing APACG with acute high IOP (20). Moreover, Saricaoglu et al. showed that high preoperative IOP and IOP fluctuations were the main risk factors for decompression retinopathy (21). This phenomenon may be attributed to the sudden reduction in IOP, which decreases retinal arterial resistance, leading to increased flow and leakage through already fragile capillaries, and induces forward movement of the LC, leading to axonal transport blockage and central retinal vein compression, causing hemorrhagic retinopathy similar to central retinal vein occlusion.

The BCVA of all patients experiencing an APAC attack partially improved after IOP control. However, at the last follow-up, the BCVA in the ODH group remained significantly lower than that in the control group (*p* < 0.001), suggesting that visual function was affected despite IOP control following APAC attacks.

In the control group, no significant difference in RNFLT was observed (*p* > 0.05) during the follow-up period, indicating that there was no distinct change in RNFLT within 12 months under normal physiological conditions. In the ODH group, the RNFLT in each sector initially thickened predominantly in the early stage post-IOP control but gradually thinned at 2 weeks, eventually stabilizing at 12 months. Previous studies have demonstrated that RNFLT predominantly increases within 2 weeks after IOP control and significantly thins from 1 month to 6 months post-APAC attack compared to that in the contralateral eyes (22, 23). This increase in RNFLT may be due to acute, persistent high IOP mechanically compressing the optic nerve, LC area, and blood vessels, resulting in LC deformation, axoplasmic flow blockage, prolonged ischemia and hypoxia, retinal ganglion cell (RGC) apoptosis, and optic nerve edema (24). Alternatively, post-IOP control-induced ischemia–reperfusion injury may cause intracellular calcium overload and excess oxygen-free radical and inflammatory factor release, exacerbating RGC apoptosis and atrophy (25).

VF assessment revealed significant MD damage, reduced visual sensitivity, and diffuse VF defects within 72-h post-IOP control during APAC. IOP control significantly improved the MD at and VF defects (*p* < 0.05) at 1 month, and this significant improvement in the MD persisted at 6 months, consistent with the findings of a previous study (26). MD decreased over time and remained stable at 12 months. Compared to the MD in the control group, the MD was still significantly reduced at 12 months in the ODH group, suggesting that acute, persistent high IOP caused visual function damage, which was partly reversed after IOP control, consistent with previous findings (27). This may be attributed to the acute high IOP causing diffuse ischemia and hypoxia in the RNFL, tissue edema, and impaired visual function. After the IOP decreased to normal, the ischemia and hypoxia improved, and the abnormal substances released as a result of the ischemia and ischemia-repercussion injury gradually decreased. The condition naturally improved, RNFL edema subsided, and visual function partially recovered.

Studies have shown that APAC attacks, the duration of the highest IOP, and RNFLT loss are associated with VF damage (26). In the study by Li et al. (28), the IOP during the acute attack was 53.97 ± 13.27 mmHg, the time from symptom appearance to treatment was 6.49 ± 9.81 days, and the proportion of blindness caused by APAC was12.54% post-treatment. This suggests that a high IOP and prolonged TST may lead to increased optic nerve damage and subsequently contribute to a higher proportion of blindness (VA < 3/60) in APAC cases (28). The results of the present study showed that the duration of the highest IOP directly impacted ODH: the longer the duration of the highest IOP, the larger the scope of ODH. In addition, we found that the scope of ODH significantly impacted the MD value (*β* = 1.97, *T* = −8.90, *p* < 0.001), that is, the larger the scope of ODH, the more severe the VF damage. Therefore, early reduction of IOP, minimizing the duration of elevated IOP, and timely effective treatment of APAC are crucial for enhancing patients’ visual function and quality of life and reducing the proportion of glaucoma-induced blindness, particularly in the Asia-Pacific region.

Liu et al. (22) reported that IOP exceeding 40 mmHg could impact the optic nerve. In our study, the average IOP among patients was 64.28 ± 10.36 (range: 56–87) mmHg, which significantly surpassed the threshold value of optic nerve damage. This may explain why the highest IOP showed a relatively weak correlation with ODH and visual function. Additionally, we noted that VF defects mainly manifested as a decrease in diffuse visual acuity.

This study had some limitations. Specifically, the sample size was small, and the follow-up time was limited. Future studies should include large sample sizes and observe ODH characteristics during APAC attacks, changes in ODH and the optic nerve after IOP control, and factors associated with ODH over a long period of time.

## 5 Conclusion

This study revealed that ODH, presenting as flame and splinter shapes, mostly occurred in the TI, TS, and T sectors of the optic disc in patients experiencing their first APAC attack. The duration of high IOP was positively correlated with the ODH scope; the larger the ODH scope, the more severe the MD damage. After IOP control, the RNFLT became swollen and then thinner, stabilized 6 months later, and the MD was partly improved. Our findings suggest that timely and effective IOP management is essential for restoring visual function following APAC.

## Data availability statement

The original contributions presented in the study are included in the article/supplementary material, further inquiries can be directed to the corresponding authors.

## Ethics statement

The studies involving humans were approved the Ethics Committee of Shijiazhuang People’s Hospital. The studies were conducted in accordance with the local legislation and institutional requirements. Written informed consent for participation was not required from the participants or the participants’ legal guardians/next of kin in accordance with the national legislation and institutional requirements.

## Author contributions

HZ: Writing – original draft, Writing – review & editing. YL: Writing – review & editing, Writing – original draft. YT: Writing – review & editing. XY: Writing – review & editing. YG: Writing – review & editing. WL: Writing – review & editing. KS: Writing – review & editing. GT: Writing – review & editing. HG: Writing – review & editing.
